# Inclisiran adjuvant therapy to statins for the use of hypercholesterolemia: a commentary

**DOI:** 10.1186/s43044-023-00389-8

**Published:** 2023-07-13

**Authors:** Laiba Imran Vohra, Kashf Rizwan, Emaan Saeed, Muhammad Syed Ali Hamza, Sidhant Ochani

**Affiliations:** 1 Department of Medicine, Ziauddin University, Karachi, Pakistan; 2 Baqai Medical University, Karachi, Pakistan; 3 Department of Medicine, Khairpur Medical College, Khairpur Mir’s, 66020 Pakistan

**Keywords:** Inclisiran, Hypercholesterolemia, Statins, Low-density lipoproteins, LDL

## Abstract

**Background:**

Hypercholesterolemia is a lipid disorder characterized by excessively high levels of low-density lipoproteins, which encourages fat accumulation in your arteries, hence escalating the chances of heart attack and stroke. Globally, 39% of individuals experience elevated total cholesterol levels with 98.6 million DALYs (disability-adjusted life years) caused by high non-HDL cholesterol in 2019, supposedly killing 4.4 million people.

**Main body:**

LDL cholesterol is the primary target of treatment for lowering the risk of cardiovascular events in both primary and secondary prevention. The usual drug to achieve this goal is HMG-CoA reductase inhibitors (statins), which constitute the most potent and effective class to reduce LDL cholesterol. The current treatment of choice for hypercholesterolemia is statin therapy; however, a considerable proportion of patients are unable to reach their desired low-density lipoproteins levels (LDL), while some cannot take statins at all. The regular use and possible non-adherence to long-term therapy of statins have prompted the development of novel PCSK9-targeting drugs such as inclisiran—a synthesized small interfering RNA. Inclisiran binds to the proprotein convertase subtilisin/kexin type 9 (PCSK9) mRNA causing its disintegration and hence preventing its formation. This results in reduced amounts of PCSK9 both within and outside the cells, which significantly lowers LDL levels. Multiple double-blind, placebo-controlled Osaka Emergency Information Research Intelligence Operation Network System (ORION) trials were conducted; ORION-9 was conducted on patients with familial hypercholesterolemia and LDL cholesterol levels higher than 100 mg/dl despite taking the maximum dose of statin therapy, whereas ORION-10 and ORION-11 were conducted on patients with cardiovascular disease or having its risk factors. These patients were administered Inclisiran injections on days 1, 90 (month 3), 270 (month 9), and 450 (month 15) and were followed for 540 days. The results showed decreased LDL levels by 51% compared to the placebo and further established a strong link with reduced major adverse cardiac events rates with no effect on creatinine kinase and liver function test levels. The drug’s significant side effect was reported to be an injection site reaction.

**Conclusion:**

Inclisiran may be utilized alone or in conjunction with other lipid-lowering treatments in individuals who are unable to take statins or for whom they are contraindicated. Furthermore, its exceptional stability throughout a broad range of heat conditions makes its use well-suited for developing countries.

## Background

Hypercholesterolemia is a lipid disorder characterized by excessively high levels of low-density lipoproteins (LDL), which encourages fat accumulation in your arteries, hence escalating the chances of heart attack and stroke [1]. Globally, 39% of individuals experience elevated total cholesterol with 98.6 million disability-adjusted life years (DALYs) caused by high non-high density lipoprotein (HDL) cholesterol in 2019, supposedly killing 4.4 million people. [2]

The second National Diabetes Survey of Pakistan (NDSP) 2016–2017 conducted a population-based sub-analysis highlighting a 39.3% prevalence of hypercholesterolemia in Pakistan, with Punjab having the highest rate (41.6%) and Baluchistan having the lowest (22.7%). A total of 10,834 participants (43.8% men and 56.2% women) with a mean age of 43.8 ± 14.0 years took part in the study, among which 39.3% of the individuals had high cholesterol, 48.9% had high triglycerides, 39.7% had high low-density lipoprotein cholesterol (LDL-C) levels, and 83.9% of the males and 90% of the women had low HDL values. The 50–59 age category had the greatest levels of high cholesterol and triglycerides, whereas the 40–49 age category had the highest prevalence of high LDL and low HDL. It was shown that diabetes, obesity, and hypertension were the main risk factors for dyslipidemia. This is mainly due to the greater financial standing of the Urban Population, lack of exercise, and poor dietary habits. [3]

## Main text

### Statin therapy for the hypercholesterolemia

LDL cholesterol is the primary target of treatment for lowering the risk of cardiovascular events in both primary and secondary prevention. The usual drug to achieve this goal is hydroxymethylglutaryl-coenzyme A (HMG-CoA) reductase inhibitors (statins), which constitute the most potent and effective class to reduce LDL cholesterol [4]. The current treatment of choice for hypercholesterolemia is statin therapy; however, a considerable proportion of patients are unable to reach their desired LDL, while some cannot take statins at all [5]. This includes individuals older than 70 years of age, those experiencing unpleasing side effects or having a history of liver disease, or those taking medications that interfere with statin therapy such as warfarin, danazol, and certain HIV medicines. [6]

### Recommended use of inclisiran for lowering LDL levels

The regular use and possible non-adherence to long-term therapy of statins have prompted the development of novel Proprotein convertase subtilisin/kexin type 9 serine protease (PCSK9)-targeting drugs such inclisiran—a synthesized small interfering RNA (siRNA). Inclisiran binds to the PCSK9 mRNA causing its disintegration and hence preventing its formation. This results in reduced amounts of PCSK9 both within and outside the cells, which significantly lowers LDL levels [5]. Preclinical studies and clinical trials have revealed a linear relationship between the drug's dosage and effectiveness. Multiple double-blind, placebo-controlled Osaka Emergency Information Research Intelligence Operation Network System (ORION) trials were conducted; ORION-9 was conducted on patients with familial hypercholesterolemia and LDL cholesterol levels higher than 100 mg/dl despite taking the maximum dose of statin therapy, whereas ORION-10 and ORION-11 were conducted on patients with cardiovascular disease or having its risk factors. These patients were administered Inclisiran injections on days 1, 90 (month 3), 270 (month 9), and 450 (month 15), and were followed for 540 days. The results showed decreased LDL levels by 51% compared to the placebo and further established a strong link with reduced major adverse cardiac events (MACE) rates with no effect on creatinine kinase (CK) and liver function test (LFT) levels. The drug’s significant side effect was reported to be an injection site reaction [5]. The US Food and Drug Association (FDA) approved the drug on 22 December 2021 for adults with heterozygous familial hypercholesterolemia or cardiovascular disease requiring further reduction of LDL levels [7]. Yet, this drug is not used in Pakistan and its usage is highly recommended.

## Conclusions

While statin treatments have been extensively researched and are strongly endorsed by randomized controlled trials and clinical guidelines, not every patient can tolerate statin therapy or attain an adequate reduction in LDL-C levels through statin treatment alone. Inclisiran provides convenience as it needs to be administered every 6 months and additionally lowers levels by 50% [8]. This will most likely contribute to its cost effectiveness and lead to better compliance [9]. Furthermore, its exceptional stability throughout a broad range of heat conditions [9] makes its use well-suited for developing countries like Pakistan.

## Data Availability

All data and materials can be requested by the corresponding author SO and can be provided as per request.

## Abbreviations

LDL: Low-density lipoproteins
DALYs: Disability-adjusted life years
HDL: High-density lipoprotein
NDSP: National Diabetes Survey of Pakistan
LDL-C: Low-density lipoprotein cholesterol
HMG-CoA: Hydroxymethylglutaryl-coenzyme A
PCSK9: Proprotein convertase subtilisin/kexin type 9 serine protease
siRNA: Small interfering RNA
ORION: Osaka Emergency Information Research Intelligence Operation Network System
MACE: Major adverse cardiac events
CK: Creatinine kinase
LFT: Liver function test
FDA: Food and Drug Association
